# Glucocerebrosidase Mutations alter the endoplasmic reticulum and lysosomes in Lewy body disease

**DOI:** 10.1111/j.1471-4159.2012.07879.x

**Published:** 2012-10

**Authors:** Marzena Kurzawa-Akanbi, Peter S Hanson, Peter G Blain, Debra J Lett, Ian G McKeith, Patrick F Chinnery, Christopher M Morris

**Affiliations:** * Medical Toxicology Centre, Wolfson Building, Newcastle UniversityNewcastle upon Tyne, UK; † Wolfson Research Centre, Institute for Ageing and Health, Newcastle UniversityNewcastle, UK; † Institute of Genetic Medicine, Newcastle UniversityNewcastle upon Tyne, UK

**Keywords:** dementia with Lewy bodies, endoplasmic reticulum, glucocerebrosidase, Lewy body disease, lysosome, Parkinson’s disease

## Abstract

Lewy body disease (LBD) development is enhanced by mutations in the *GBA* gene coding for glucocerebrosidase (GCase). The mechanism of this association is thought to involve an abnormal lysosomal system and we therefore sought to evaluate if lysosomal changes contribute to the pathogenesis of idiopathic LBD. Analysis of post-mortem frontal cortex tissue from 7 *GBA* mutation carriers with LBD, 5 *GBA* mutation carriers with no signs of neurological disease and human neural stem cells exposed to a GCase inhibitor was used to determine how *GBA* mutation contributes to LBD. *GBA* mutation carriers demonstrated a significantly reduced level of GCase protein and enzyme activity and retention of glucocerebrosidase isoforms within the endoplasmic reticulum (ER). This was associated with enhanced expression of the lysosomal markers LAMP1 and LAMP2, though the expression of ATP13A2 and Cathepsin D was reduced, along with the decreased activity of Cathepsin D. The ER unfolded protein response (UPR) regulator BiP/GRP78 was reduced by *GBA* mutation and this was a general phenomenon in LBD. Despite elevation of GRP94 in LBD, individuals with *GBA* mutations showed reduced GRP94 expression, suggesting an inadequate UPR. Finally, human neural stem cell cultures showed that inhibition of GCase causes acute reduction of BiP, indicating that the UPR is affected by reduced glucocerebrosidase activity. The results indicate that mutation in *GBA* leads to additional lysosomal abnormalities, enhanced by an impaired UPR, potentially causing α-synuclein accumulation.

Several lines of evidence indicate that mutations in the *GBA* gene coding for glucocerebrosidase (Glucosylceramidase, GCase) contribute to the development of Parkinson’s disease (PD) and dementia with Lewy bodies (DLB); however, the underlying mechanism is still to be established. Whilst there is a variable strength of association in certain populations, a large meta-analysis has demonstrated the odds ratio for any *GBA* mutation in patients versus controls to be 5.43 across centres, providing validation of *GBA* as a risk gene for PD (Sidransky *et al.* 2009). *GBA*-associated parkinsonism seems to be characterized by an earlier onset and an increased likelihood of cognitive decline and dementia, compared with typical PD (Neumann *et al.* 2009; Sidransky *et al.* 2009; Seto-Salvia *et al.* 2012). Features typical of DLB also cluster with *GBA*-associated phenotypes (Goker-Alpan *et al.* 2008) and population studies have shown an increased frequency of *GBA* mutations in DLB as in PD (Goker-Alpan *et al.* 2006; Mata *et al.* 2008). DLB, PD and PD with dementia (PDD) belong to the common spectrum of Lewy body disease (LBD), as they are characterized by the overlapping clinical symptoms reflecting the common pathogenesis, i.e. the dismetabolism of α-synuclein and formation of Lewy bodies in the brain (Lippa *et al.* 2007). Mutant GCase, therefore, may be a contributory factor to the development of Lewy body disease in general.

Homozygous mutations in the *GBA* gene cause Gaucher’s disease (GD), the most prevalent lysosomal storage disorder, due to deficiency of glucocerebrosidase activity, and the accumulation of its main substrate glucocerebroside (d-glucosyl-N-acylsphingosine, glucosylceramide) in lysosomes in macrophages (Barranger and Ginns 1989). GD patients present a wide spectrum of clinical phenotypes with three subtypes of GD; type 1–non-neuronopathic (MIM:230800), type 2–acute neuronopathic (MIM:230900) and type 3–subacute neuronopathic (MIM:2301000) with involvement of the nervous system in type 2 and 3, in contrast to type 1 (Barranger and Ginns 1989). Early-onset parkinsonism has also been associated with GD. Initial reports described mild late-onset GD patients with typical PD that included tremor, bradykinesia, rigidity and often cognitive decline, with a poor response to conventional anti-parkinsonian therapy (Neudorfer *et al.* 1996), with typical neuropathological changes (Wong *et al.* 2004). A family history of parkinsonism in GD probands has also been reported, demonstrating the potential predisposition of *GBA* heterozygotes to parkinsonism (Goker-Alpan *et al.* 2004). The presence of glucocerebrosidase in Lewy bodies and Lewy neurites in patients carrying *GBA* mutations suggests that glucocerebrosidase can contribute to the aggregation of α-synuclein and Lewy body formation (Goker-Alpan *et al.* 2010). Increased α-synuclein immunoreactivity has been observed in cells treated with conduritol B epoxide (CBE), an inhibitor of GCase activity, along with accumulation of α-synuclein within the substantia nigra in CBE-treated mice (Manning-Bog *et al.* 2009). This indicates that reduced glucocerebrosidase activity may promote alterations in α-synuclein biology and these changes are also present in the brains of mice and in neuronal cells carrying *GBA* mutations (Cullen *et al.* 2011; Mazzulli *et al.* 2011; Sardi *et al.* 2011). It is plausible that mutant glucocerebrosidase interferes with cellular clearance of α-synuclein via lysosomal pathway and stimulates protein aggregation (Cuervo *et al.* 2004; Mazzulli *et al.* 2011), thus predisposing to the development of synucleinopathies.

To gain an insight into the effects of glucocerebrosidase on neuronal function, we aimed to evaluate the effects of mutant *GBA* gene in human frontal cortex tissue by examining glucocerebrosidase protein levels and activities in *GBA* mutation carriers and establishing whether lysosomal changes were evident. Furthermore, a hypothesis, that the presence of misfolded proteins, perhaps glucocerebrosidase, in the ER induces ER stress and contributes to the development of LBD, was also explored.

## Materials and methods

### Case control analysis

All procedures were approved by the Local Research Ethics Committee. A prospective clinicopathological series of patients with DLB (*n* = 105) or PD (*n* = 79) fulfilling clinical and neuropathological criteria (Gibb and Lees 1988; McKeith *et al.* 1996; McKeith 2006) was screened for mutations in *GBA*. PD and DLB cases were grouped together as LBD since pathological changes were similar, clinical presentation differing only in the timing of any dementia in relation to PD. A cohort of elderly normal individuals (*n* = 164 pathologically confirmed) without a history of neurological or psychiatric impairment was also genotyped and used as a control population. DNA was extracted from frozen post-mortem brain tissue using standard methods. Seven specific mutations in *GBA* were identified and screened using PCR-RFLP-based assays and cycle sequencing (see Table 1 for case details and Table S1).

**Table 1:** Case details of mutation-carrying donor samples

Patient	*GBA* mutation	Clinical diagnosis	Gender	AAO-Dem (years)	AAO-PD (years)	Age-Death (years)
DLB_1	IVS2 + 1	DLB	Female	65	Not Identified^a^	71
DLB_2	RecNciI	DLB	Female	51	52	57
DLB_3	N370S	DLB	Female	74	79	82
DLB_4	L444P	DLB	Female	75	75	77
PD_1	IVS2 + 1	PDD	Male	73	70	74
PD_2	L444P	PD	Male	No dementia	66	70
PD_3	L105R	PDD	Female	76	Not known	78
C_1	RecNciI	Control	Female	NA	NA	89
C_2	N370S	Control	Female	NA	NA	86
C_3	L444P	Control	Female	NA	NA	83
C_4	N370S	Control	Male	NA	NA	55
C_5	N370S	Control	Male	NA	NA	51

a For case DLB_1, no clinical details indicating parkinsonism could be identified on the case note review. Pathology showed widespread substantia nigra neuron loss and cortical Lewy bodies.

AAO-Dem, Age at onset of dementia where this could be accurately ascertained; AAO-PD, Age at onset of parkinsonism movement disorder where this could be accurately determined; NA, not applicable.

### Tissue analysis

Post-mortem frontal cortex (Brodmann Area 8) tissue was used as an area relatively spared from major LBD pathology and cell loss, which may confound studies in regions such as substantia nigra. *GBA* mutation being present in all cells would be expected to have effects in most cell types. Tissue from individuals identified from the cohort as carrying *GBA* mutations (4 DLB, 3 PD and 5 controls) and non-carriers (7 DLB, 8 PD and 11 controls) was rapidly thawed and approximately 250 mg of grey matter homogenized over ice using a rotor stator-type homogenizer in 10 volumes of 0.32 M sucrose, 1 mM EDTA, 50 mM Tris, pH 7.4 (Sigma, Gillingham, Dorset, UK), protease inhibitor cocktail tablets EDTA-free (Roche, Burgess Hill, West Sussex, UK) containing buffer. After addition of Triton X-100 (Sigma) to a final concentration of 1%, samples were sonicated for 20 min on ice in a sonicating bath. Another subset of four human spleen and four frontal cortex tissue samples (30–180 mg wet tissue) were homogenized as previously. Triton X-100 was then added to a final concentration of 1%, followed by incubation on ice for 30 min and centrifugation at 25 200 *g* for 60 min. Supernatants were subjected to the further analysis as Triton X-100-soluble fractions. Protein concentration was determined using Bradford assay.

### GCase enzymatic activity

To investigate the effects of *GBA* mutation on brain enzyme activity, a standard glucocerebrosidase activity assay at acidic pH was employed. Residual enzyme activity was determined as the production of fluorescent 4-methylumbelliferone (4-MU) from 4-methylumbelliferyl-β-D-glucopyranoside (4-MUG) substrate as described previously (Takagi *et al.* 1999). Sample triplicates, each containing 50 μg protein (brain homogenates) or 10 μg protein (cell lysates) were diluted in assay buffer of 50 mM citrate phosphate buffer, pH 5.0 and 0.25% sodium taurocholate (Sigma) containing 0.5 mM 4-MUG in 96-well black polystyrene plates. The reactions were incubated at 37°C for 1–2 h and terminated by addition of 200 μL of 200 mM carbonate-bicarbonate buffer, pH 10.5. Fluorescence was measured (Ex 360 nm, Em 460 nm) and a 4-MU standard solution (0–4000 nM) was used to generate a standard curve. Control of glucocerebrosidase inhibition was carried out with the addition of 100 μM of the specific glucocerebrosidase inhibitor CBE (Sigma), which reduced activity to approximately 15% of total values.

### Western blot analysis

Samples of brain tissue homogenates and cell lysates containing 10 μg of protein were resolved by electrophoresis on 10% or 4–12% sodium dodecyl sulphate–polyacrylamide gels (Invitrogen, Paisley UK), and transferred to nitrocellulose membranes using iBlot Gel Transfer Stacks (Invitrogen). The membranes were probed with primary and secondary antibodies (see Table S2) using standard methods and developed using Amersham ECL detection reagents (GE Healthcare, Buckinghamshire, UK) and exposed to autoradiography film (Kodak, Sigma, Gillingham, Dorset, UK). The blots were scanned and band intensities were analysed using ImageJ software. Glyceraldehyde 3-phosphate dehydrogenase (GAPDH) was used to normalize the results and the ratios of mean protein/GAPDH were calculated. The results are presented as mean ± SD.

### Endo-H and PNGase F treatment

To determine the effects of *GBA* mutation on glucocerebrosidase glycosylation and oligosaccharide chain development, either Endoglycosidase H, to strip proteins of high mannose oligosaccharides [complex oligosaccharides are resistant to Endoglycosidase-H (Endo-H) activity], or Peptide N glycosidase F (PNGase F), to remove all types of asparagine N-linked oligosaccharides, was used. Frontal cortex tissue homogenates or spleen and frontal cortex Triton X-100-soluble fractions, containing 20 μg of protein, were treated with either PNGase F or Endo-H (New England Biolabs, Hitchin, Herts., UK) according to the manufacturer’s instructions. Treated samples and non-treated controls were resolved using 4–12% sodium dodecyl sulphate–polyacrylamide gels (Invitrogen) and analysed by Western blot.

### Cathepsin D enzymatic activity

The residual Cathepsin D activity was measured in the human brain tissue samples using a kinetic assay (Yasuda *et al.* 1999). Cathepsin D substrate solution (Enzo Life Sciences, Exeter, UK) was used at a final concentration of 20 μM. Pepstatin A (Enzo Life Sciences) solution at a final concentration of 0.2 mg/mL was used for the selective inhibition of Cathepsin D. Briefly, 5 μg of protein samples (prepared in native lysis buffer containing 0.32 M Sucrose, 1 mM EDTA, 50 mM Tris, pH 7.4 (all Sigma) and EDTA-free protease inhibitor cocktail tablets (Roche)) were diluted in an assay buffer (50 mM sodium acetate buffer, pH 4.0 (Sigma)) in a final volume of 10 μL. In 96-well black polystyrene plates, 40 μL of the assay buffer or for the control samples with inhibitor, 40 μL of Pepstatin A solution in the assay buffer were added to the protein samples and pre-incubated at 37°C for 10 min and subsequently, 50 μL of the substrate solution in the assay buffer pre-heated to 37°C were added to all samples. The increase in fluorescence was measured (Ex 320 nm, Em 400 nm, sensitivity 100, 37°C). For 30-min incubation, the readings were taken at intervals of 5 min. 7-methoxycoumarin-4-acetic acid (Sigma) in the assay buffer (0–1.5 nmoles (0–15 μM)) was used to generate a standard curve. Pepstatin A caused a complete inhibition of enzyme activity.

### Human neural stem cell cultures

Human neural precursor stem cell lines were grown as neurospheres according to previously described methods (Burnstein *et al.* 2004) at 37°C in a 95% air/5% CO_2_, humidified incubator. Proliferation medium was replenished at 2–3-day intervals by replacing 60–70% of the medium with fresh medium. For assay, cells were extensively triturated to small neurospheres and seeded into 25 cm flasks coated with 0.5% gelatine (Sigma) and grown for 10 days. Growth medium was replaced with differentiating medium containing Dulbecco’s modified Eagle’s medium /F12 supplemented with 10% heat-inactivated Foetal Bovine Serum (Sigma), N-1, B27 and N-2. Conversion of neurospheres into cells with neural morphology took 2–3 days. Cells were allowed to grow for a further 14 days to develop neuritic networks after which they were treated for 72 h with 100 μM CBE or dimethyl sulfoxide (0.02%) vehicle. Cells were lysed using ice-cold native lysis buffer [Tris-buffered saline containing 0.32 M Sucrose, 1% Triton X-100 (all Sigma), 1 × protease/phosphatase inhibitor cocktail (Roche)] and sonicated for 20 min on ice in a sonicating bath. Subsequently, lysates were centrifuged at 1000 *g* for 10 min and the supernatants subjected to Western blotting. Protein concentration was determined using Bradford assay. Data are the mean of three independent replicates.

### Statistical analysis

*F*-test two-sample for variances and *t*-test: two-sample assuming equal or unequal variances were used to assess statistical significance of the enzyme activity and Western blot analysis data. For the stem cell culture studies, the data consist of the mean of all three independent replicates and the paired sample statistics of the three independent experiments was performed using spss Statistics 17.0 (SPSS, Chicago, IL, USA). Statistical significance was considered when *p* two-tail ≤ 0.05. Graphs were generated using GraphPad Prism 4 software (GraphPad, San Diego, CA, USA). The results are presented as mean ± SD. Fold changes (FC) represent: average of ratios of mean (protein/GAPDH) for treated (mutant) samples/average of ratios of mean (protein/GAPDH) for untreated (control) samples.

## Results

### GCase activity

**Fig 1:**
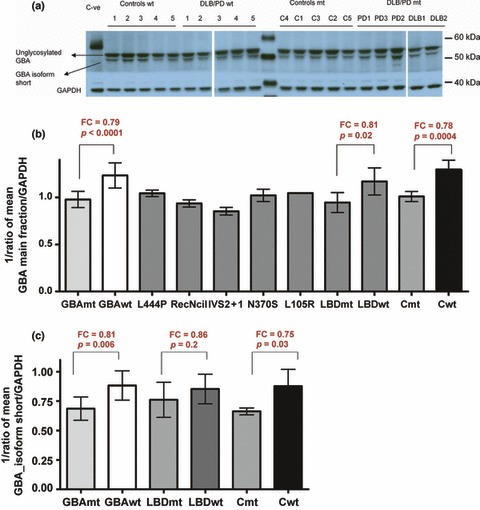
Peptide N-glycosidase F (PNGase F)-treated glucocerebrosidase in normal and *GBA* mutation carriers: Brain homogenates were treated with PNGase F to show protein molecular weight and Western blotting for glucocerebrosidase used to detect glucocerebrosidase protein (a). The major isoform of glucocerebrosidase was reduced in *GBA*-mutation heterozygotes (*GBA* mutations: L444P, RecNciI, IVS2 + 1 G>A, N370S and L105R) (LBDmt, *n* = 5; Cmt, *n* = 5) compared to wild-type *GBA* individuals (LBDwt, *n* = 5; Cwt, *n* = 5) (b). Reductions in the major isoform were seen irrespective of the presence of Lewy body disease. Changes were seen in the levels of the short isoform of glucocerebrosidase in controls with *GBA* mutations, although this was not significant in LBD cases and *GBA* mutation carriers; overall, the short isoform was significantly reduced (c). Figures are Mean GBA/GAPDH ratio ± SD. Numbering of mutation bearing cases is according to Table 1.

**Fig 2:**
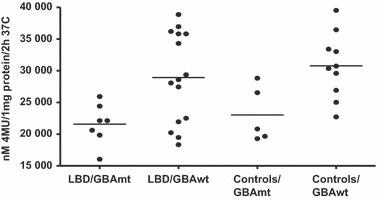
Brain glucocerebrosidase enzyme activity is decreased in *GBA* mutation carriers: Samples of grey matter (LBDmt *n* = 7, LBDwt *n* = 15, Cmt *n* = 5, Cwt *n* = 10) were assayed for glucocerebrosidase enzyme activity using the specific substrate and levels of 4-methylumbelliferone (4-MU) production were determined. Comparison with tissue from individuals with wild-type *GBA* were used as control and showed reduced glucocerebrosidase enzyme activity in *GBA* mutation carriers. Glucocerebrosidase activity was reduced in both normal and Lewy body disease (LBD) cases to a similar extent. Note that there is an overlap in glucocerebrosidase activity between control and mutant samples.

Brain tissue from the frontal cortex of LBD cases and controls, both with and without *GBA* mutation, were screened for the relative levels of glucocerebrosidase protein by Western blotting (Fig 1). Analysis of relative levels of glucocerebrosidase protein (following PNGase F treatment) showed a reduction in the longer isoform of glucocerebrosidase of approximately 20% in mutation carriers (*p* < 0.05, mutant vs. wild type, LBDmt *n* = 5, LBDwt *n* = 5, Cmt *n* = 5, Cwt *n* = 5), irrespective of disease state, with the shorter isoform showing a reduction in levels in the control samples carrying mutation (*p* < 0.05, mutant vs. wild type), although this was not significantly reduced in LBD cases (*p* > 0.05, mutant vs. wild type, Fig 1). Alterations in GCase were not because of either neuronal loss or gliosis as we found no significant change in NeuN or GFAP between samples (not shown). This was confirmed by analysis of glucocerebrosidase enzyme activity, which again showed an approximately 25% reduction in enzyme activity in *GBA* mutation carriers irrespective of disease state (Fig 2), with no reduction in activity because of the presence of LBD. Individual analysis of specific mutations did not show any consistent pattern of enzyme activity reduction (not shown), although we cannot rule out a genotype–phenotype relationship, given the reduced numbers of cases studied.

### Lysosomal markers

**Fig 3:**
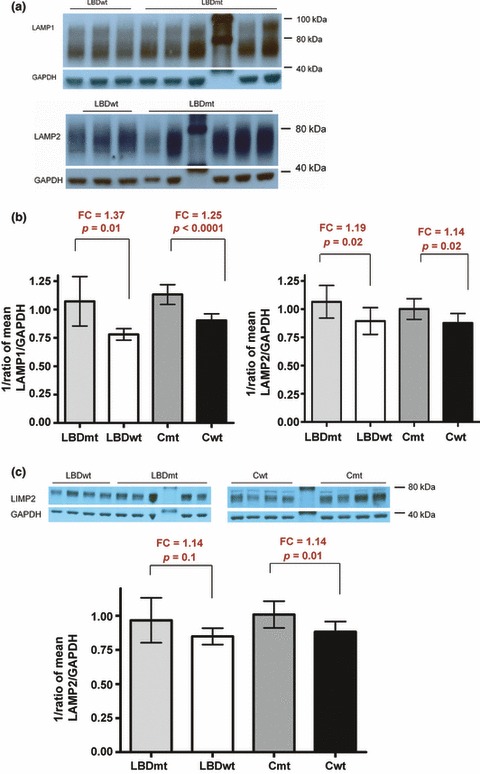
Major lysosomal membrane proteins are elevated in *GBA* mutation carriers: Western blotting was used to determine the relative levels of LAMP1, LAMP2 and LIMP-2 protein in brain homogenates from individuals with and without *GBA* mutations (LBDmt *n* = 5–7, LBDwt *n* = 14–15, Cmt *n* = 5, Cwt *n* = 10–11)(a, c). Elevated levels of LAMP1 and LAMP2 were seen in Lewy body disease (LBD) and normal individuals in the presence of *GBA* mutation (b). The major protein involved in import of glucocerebrosidase into lysosomes LIMP-2 (SCARB2) was also seen to be elevated in control mutation carriers (c) but was not significantly elevated in LBD mutation carriers. Error bars are ± SD.

**Fig 4:**
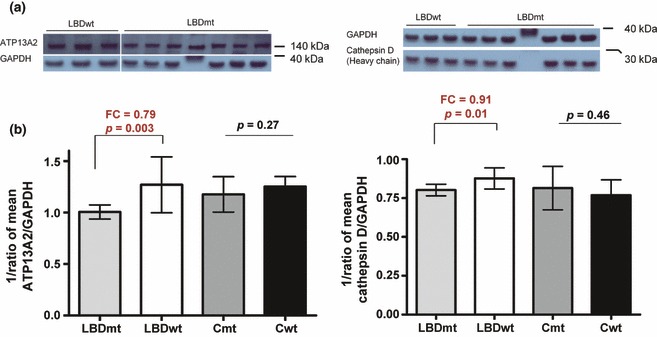
Selective alterations in lysosomal proteins in *GBA* mutation carriers with Lewy body disease: Brain tissue from *GBA* mutation carriers was screened for levels of the Kufor–Rakeb Parkinsonism protein ATP13A2 and the lysosomal protease Cathepsin D by Western blotting (LBDmt *n* = 7, LBDwt *n* = 14, Cmt *n* = 5, Cwt *n* = 11) (a) and relative levels determined against GAPDH levels. Reduced levels of ATP13A2 were seen in LBD cases carrying *GBA* mutations but not in normal individuals with *GBA* mutation (b) and similar reductions in Cathepsin D heavy chain (28 kDa) were seen associated selectively with LBD patients. Error bars are ± SD.

As glucocerebrosidase is a major lysosomal enzyme, we tested whether the presence of reduced glucocerebrosidase levels as a result of *GBA* mutation causes any change in lysosomal composition. Using the major lysosomal membrane protein (LAMP1) as a marker of lysosome mass (Eskelinen 2006), an elevation of LAMP1 associated with the presence of *GBA* mutation was seen in both normal individuals (*p* < 0.05, Cmt *n* = 5 vs. Cwt *n* = 10), and in individuals with LBD (*p* < 0.05, LBDmt *n* = 7 vs. LBDwt *n* = 15), Fig 3). Similarly, LAMP2 relative protein levels were also found to be elevated by approximately 10–15% in *GBA* mutation carriers (*p* < 0.05, LBDmt *n* = 5 vs. LBDwt *n* = 14; *p* < 0.05 Cmt *n* = 5 vs. Cwt *n* = 10). As glucocerebrosidase uses LIMP-2/SCARB2 for import and sequestration by lysosomes (Reczek *et al.* 2007) the effects of *GBA* mutation on expression were determined. Control cases carrying *GBA* mutation showed significantly increased LIMP-2 expression (*p* < 0.05, Cmt *n* = 5 vs. Cwt *n* = 11), and whilst increased, this was not significant in LBD cases with *GBA* mutation (0.05 < *p* < 0.1, LBDmt *n* = 7 vs. LBDwt *n* = 15), Fig 3). Whilst the elevation of these major lysosomal proteins pointed towards a general increase in lysosomal mass, not all lysosomal proteins were increased. Specifically, the Parkinsonism-related protein ATP13A2, involved with Kufor–Rakeb syndrome, was reduced in LBD *GBA* mutation carriers (*p* < 0.05, LBDmt *n* = 7 vs. LBDwt *n* = 14), see Fig 4), as was the major lysosomal protease Cathepsin D (Fig 4) in LBD cases (*p* < 0.05, LBDmt *n* = 7 vs. LBDwt *n* = 14), but not in controls carrying *GBA* mutation (*p* > 0.05, Cmt *n* = 5 vs. Cwt *n* = 11). Consistently, LBD cases with *GBA* mutations showed significantly decreased activities of Cathepsin D enzyme compared with non-carriers (15% depletion, *p* < 0.05, LBDmt *n* = 7, LBDwt *n* = 15) (Figure S1). This was, however, not characteristic for control individuals with *GBA* mutations (*p* > 0.05, not significant, Cmt *n* = 5, Cwt *n* = 12). For the individuals with no alterations in *GBA*, LBD patients had significantly lower Cathepsin D activities compared with controls (16% depletion, *p* < 0.05), indicating deficiency may be more associated with LBD.

As the majority of lysosomal proteins show extensive post-translational modification, and in particular the incorporation of major carbohydrate chains which aid in protein folding and resistance to lysosomal hydrolases, we sought to determine how glucocerebrosidase is glycosylated and the status of the endoplasmic reticulum activity in *GBA* mutation carriers. The glycosylation state of glucocerebrosidase was investigated using Endo-H and PNGase F treatment to remove high-mannose carbohydrate chains and all asparagine N-linked oligosaccharides respectively. PNGase F treatment of glucocerebrosidase showed the presence of two molecular weight species of glucocerebrosidase (see Fig 1) with evidence of glycosylation of approximately 10–15 kDa. Frontal cortex tissue samples from *GBA* mutation carriers showed significantly less cross-reacting material and little evidence of resistance to Endo H treatment compared with samples of human spleen, suggesting the presence of less extensive glycosylation pattern on brain glucocerebrosidase (see Figure S2).

### Endoplasmic reticulum markers

**Fig 5:**
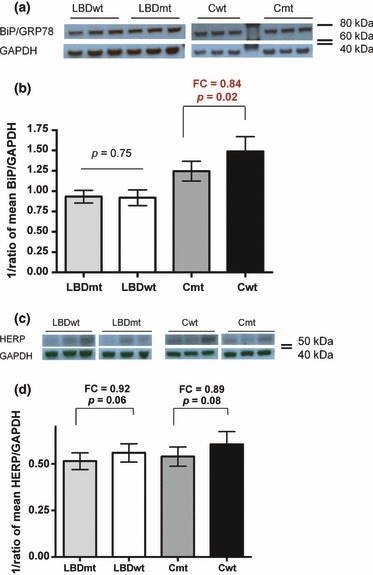
The ER Unfolded Protein Response (UPR) is abnormal in *GBA* mutation carriers: The levels of ER proteins BiP/GRP78 and HERP were determined in brain homogenates from individuals with Lewy body disease (LBD) and in normal control individuals with and without *GBA* mutations (LBDmt *n* = 7, LBDwt *n* = 13–15, Cmt *n* = 5, Cwt *n* = 10–11) (a, c). Relative levels of BiP/GRP78 were reduced in LBD cases, independent of mutation status, compared to controls (*p* < 0.001) by approximately 10–15% but reduced in control individuals (Cmt) carrying *GBA* mutation (b). HERP, a stress-induced ER protein was similarly reduced in control and LBD *GBA* mutation carriers (d) although there was no change between LBD and control samples overall (*p* = 0.3). Error bars are ± SD.

**Fig 6:**
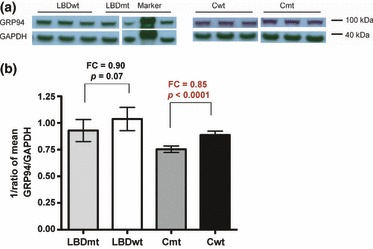
The chaperone GRP94 is elevated in Lewy body disease (LBD) but reduced by *GBA* mutation: Brain tissue from LBD and control individuals was probed for the major ER chaperone protein GRP94 by Western blotting (LBDmt *n* = 5, LBDwt *n* = 13, Cmt *n* = 4, Cwt *n* = 11) (a). Compared to controls (b) there was an elevation of GRP94 in LBD brain of approximately 15% (*p* < 0.001) suggesting the presence of an UPR, though this was reduced by the presence of *GBA* mutation in controls and to a lesser extent in LBD. Error bars are ± SD.

**Fig 7:**
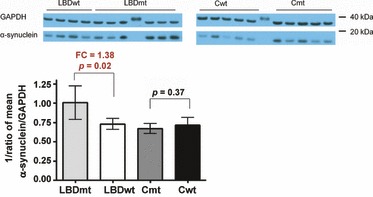
α-synuclein is elevated in frontal cortex of *GBA* mutation carriers: Increased α-synuclein protein expression is only observed in LBD patients with heterozygous *GBA* mutations and does not appear to be elevated in normal controls with *GBA* mutations (total brain tissue homogenates were used in the analysis) (LBDmt *n* = 6, LBDwt *n* = 11, Cmt *n* = 5, Cwt *n* = 11).

As there is little Endo-H-resistant glucocerebrosidase fraction in brain which may indicate ER retention and reduced processing of glucocerebrosidase, it is possible that ER marker proteins are altered by the presence of *GBA* mutation. Western blot analysis of the major UPR protein BiP/GRP78 showed a marked reduction in LBD which was independent of *GBA* mutation status, suggesting that LBD shows an abnormal UPR (*p* < 0.05, LBD vs. Control, LBDmt *n* = 7, LBDwt *n* = 15, Cwt *n* = 10). Analysis of control cases with *GBA* mutations showed a similar reduction of BiP/GRP78 to LBD, indicating the presence of an abnormal UPR caused by *GBA* mutation (*p* < 0.05, Cmt *n* = 5 vs. Cwt *n* = 11), with evidence of slightly reduced HERP (*p* < 0.1 > 0.05, LBDmt *n* = 7 vs. LBDwt *n* = 13; *p* < 0.1 > 0.05, Cmt *n* = 5 vs. Cwt *n* = 10), Fig 5). In contrast, the ER chaperone GRP94 was elevated in LBD compared with controls, though this was reduced in LBD by the presence of *GBA* mutation (*p* = 0.07, LBDmt *n* = 5 vs. LBDwt *n* = 13) and also in controls carrying *GBA* mutation (*p* < 0.05, Cmt *n* = 4 vs. Cwt *n* = 11) (Fig 6). Analysis of α-synuclein levels in total brain tissue homogenates showed that the monomeric form of the protein was elevated in LBD cases carrying *GBA* mutations compared with wild-type LBD cases (*p* < 0.05 LBDmt *n* = 6 vs. LBDwt *n* = 11; Fig 7), whilst no alterations were observed within controls (Cmt *n* = 5, Cwt n = 11).

### Cell culture analysis of GBA inhibition

**Fig 8:**
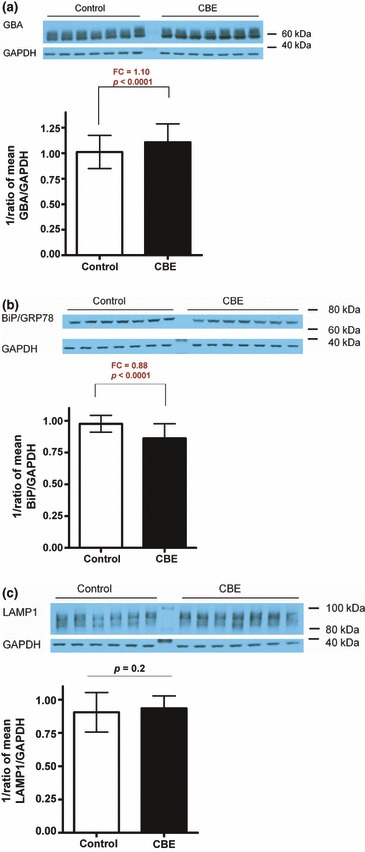
Effects of glucocerebrosidase inhibition in human neural cultures using CBE: Differentiated human neural stem cells were exposed to 100 μM of the specific glucocerebrosidase inhibitor conduritol Beta epoxide (CBE) for 72 h and protein extracts prepared for western blotting. CBE caused a slight elevation of steady-state levels of glucocerebrosidase (a) but caused an acute change of ER stress and the UPR as evidenced by reduced levels of BiP (b). Overall, no significant change was seen in the major lysosomal protein LAMP1 (c). Example blots are representative of three independent experiments. The data presented consist of the mean of all three independent replicates. Error bars are ± SD.

To determine if changes identified in the brains of *GBA* mutation carriers were attributable directly to reduced glucocerebrosidase activity, differentiated human cortical neural stem cells were treated with the glucocerebrosidase inhibitor, CBE. Cell extracts from treated cells showed a slight but significant increase in glucocerebrosidase protein (*p* < 0.05; Fig 8), although glucocerebrosidase activity was almost completely inhibited (1.8% ± 0.9 of control). Acute reduction of glucocerebrosidase activity caused a small, however significant reduction of BiP/GRP78 protein (*p* < 0.05; Fig 8), indicating similar reductions in the UPR response to that found in tissue samples, although significant lysosomal protein changes were not seen and no alteration in α-synuclein was observed (data not shown).

## Discussion

Previous studies in several populations have demonstrated the association between *GBA* mutations and Lewy body disease (Sidransky *et al.* 2009). The results of this study indicate that *GBA* heterozygotes show reduced activities of glucocerebrosidase enzyme in the frontal cortex tissue irrespective of disease state, consistent with previous reports (Choi *et al.* 2011; Mazzulli *et al.* 2011). This reduction in GCase enzyme activity is relatively mild, approximately 25% in both cases and controls carrying *GBA* mutation, and would suggest that if glucocerebrosidase activity does influence development of LBD, it does so relatively efficiently or shows a subtle effect in concert with other mechanisms. Furthermore, the results show a slight depletion of GCase protein in mutation carriers, with the main isoform significantly reduced in controls and LBD cases with *GBA* mutations; however, the significant reduction of the shorter isoform was detected only in control individuals with *GBA* mutation. Importantly, in neural tissue from LBD cases and control subjects, glucocerebrosidase appears to show little in the way of extensive glycosylation, as evidenced by almost complete sensitivity to Endo-H treatment in contrast to tissues such as spleen. This finding of different patterns of glucocerebrosidase activity and processing is not new (Pentchev *et al.* 1978; Ginns *et al.* 1982), but the finding of a reduced glycosylation in the brain may have important implications for enzyme function. Normally, glycosylation has the effect of enhancing the folding and solubility of a protein (Helenius and Aebi 2001) and in the context of lysosomally directed proteins, of enhancing protein half-life by protecting it from lysosomal proteases (Kundra and Kornfeld 1999). An intriguing possibility is that the preferential effects of *GBA* mutation on the CNS in GD on the CNS (Sidransky 2004) may be a combination of enhanced GCase sensitivity to lysosomal degradation because of reduced glycosylation along with a reduced enzyme activity because of mutation. Whilst enzyme activity shows a relatively small change, it suggests that restoration of activity may be a therapeutic option, particularly if blood–brain barrier-penetrating small molecules affecting glucocerebrosidase and also glucosylceramide synthase, to reduce substrate accumulation, could be used (Mu *et al.* 2008; Khanna *et al.* 2010; Marshall *et al.* 2010; Marugan *et al.* 2011).

Whilst the enzyme defect in glucocerebrosidase in heterozygous carriers is modest, there appear to be additional changes within the endoplasmic reticulum and lysosomes. The enhanced expression of LAMP1 and LAMP2 along with the glucocerebrosidase carrier protein LIMP-2 indicates an abnormal cellular lysosomal activity. The presence of abnormal lysosomal processing and evidence of autophagy associated with synucleinopathies is a relatively common finding (Cuervo *et al.* 2004; Chu *et al.* 2009; Dehay *et al.* 2010). Whilst the enhanced expression of LAMP1 and LAMP2 might be a response to remove aggregated proteins and damaged organelles (because of for example α-synuclein accumulation), this appears to be inefficient as evidenced by the reduced Cathepsin D levels and activities. The loss of lysosomal protease activity measured as reduced Cathepsin D and ATP13A2 protein selectively in LBD cases carrying mutation may indicate why only some mutation carriers develop LBD. Several studies have shown that reduced lysosomal Cathepsin D activity can lead to α-synuclein accumulation as can ATP13A2 reduction (Qiao *et al.* 2008; Chu *et al.* 2009; Cullen *et al.* 2009; Gitler *et al.* 2009) and it is possible therefore that lysosomal changes contribute to LBD development. Given that elevated lysosome expression is evident in normal individuals heterozygous for *GBA* mutations, it is possible that the lysosomal defect predisposes individuals to disordered protein metabolism associated with ageing, such as α-synuclein processing. The development of LBD in individuals with heterozygous *GBA* mutations may be because of additional factors such as enhanced α-synuclein expression (Chu and Kordower 2007; Satake *et al.* 2009; Simon-Sanchez *et al.* 2009) and/or inefficiency of protein degradation machinery (Collier *et al.* 2011). The changes identified in the endoplasmic reticulum markers of the UPR in *GBA* mutation carriers and in LBD may be an indication of abnormal protein processing associated with α-synuclein dismetabolism (Cooper *et al.* 2006; Hoozemans *et al.* 2007). The abnormal UPR in *GBA* mutation carriers, a finding previously noted in animal and cell models of GD (Wei *et al.* 2008), indicates that *GBA* mutation predisposes cells to abnormal protein folding, exacerbated perhaps by α-synuclein accumulation (Cullen *et al.* 2011; Sardi *et al.* 2011). The reduced levels of BiP/GRP78 seen in mutation carriers and in CBE-treated cells may predispose to neuronal cell loss as selective knockout of BiP/GRP78 in Purkinje cells causes neuronal cell death (Wang *et al.* 2010). Similarly, the failure to mount an adequate UPR in *GBA* carriers evidenced by GRP78 and GRP94 reductions may indicate a predisposition to abnormal protein folding in *GBA* mutation carriers. The current cell studies show that acute inhibition of glucocerebrosidase function leads to ER stress and reduced levels of BiP, although no changes in monomeric α-synuclein levels were detected. The present data corresponds with the previous report by Cullen *et al.*, where similar concentrations of CBE were used to inhibit GCase activity in PC12-SNCA cell lines, with no corresponding α-synuclein deposition (Cullen *et al.* 2011). In contrast, the initial findings by Manning-Bog *et al.* demonstrated elevated levels of α-synuclein following CBE exposure in SH-SY5Y cells differentiated to the neuronal phenotype and in the substantia nigra of CBE-treated mice (Manning-Bog *et al.* 2009). The predisposition of cells carrying GCase defect to synucleinopathy might be therefore a combined effect of the presence of mutant GCase protein and the loss of its enzymatic activity. It is possible that reduced GCase activity causes ER stress and UPR induction through abnormal lysosomal activity, which then exacerbates α-synuclein accumulation (Mazzulli *et al.* 2011). Alternatively, the abnormal UPR in *GBA* mutation carriers identified in this study might be caused by the enduring production of mutant GCase protein, enhanced by ageing-related impairment of the ER chaperone systems (Nuss *et al.* 2008), which may impede protein folding and degradation (Ron *et al.* 2010). Prolonged activation of the UPR leads to cell death (Tabas and Ron 2011), with nitric oxide-mediated protein damage and polyubiquitination of neuronal components being contributory factors (Uehara *et al.* 2006), all hallmark features of LBD.

In summary, the findings of this study indicate that the presence of glucocerebrosidase mutation rather than reduced GCase activity may predispose neurons to the accumulation of misfolded proteins through lysosomal abnormalities and defects in the endoplasmic reticulum. Whilst on their own, these changes do not appear to be associated with LBD, such abnormalities may underlie the vulnerability of neurons to α-synuclein dismetabolism. The small number of *GBA* mutation-bearing LBD patients and controls analysed in this study should be acknowledged and it may represent a limitation factor, although various approaches were used to investigate the potential biochemical defect in *GBA* heterozygotes. It becomes apparent that LBD may be associated with a wider spectrum of lysosomal storage disorders than GCase pathology alone (Shachar *et al.* 2011; Winder-Rhodes *et al.* 2012), implying that more general cellular mechanisms, as presented herein, may be involved.

## Abbreviations

CBE: conduritol B epoxide
DLB: Dementia with Lewy bodies
Endo-H: endoglycosidase-H
ER: endoplasmic reticulum
*GBA*: Glucocerebrosidase gene
GD: Gaucher’s disease
LBD: Lewy body disease
PD: Parkinson’s disease
PNGase F: peptide N glycosidase F
UPR: unfolded protein response

## Supporting information

**Figure S1.** Residual Cathepsin D activities in*GBA* mutation carriers.

**Figure S2.** Endoglycosidase-H sensitivity ofglucocerebrosidase species in *GBA* mutation Carriers.

**Table S1.** Patient demographics of post mortem cohort

**Table S2.** Primary antibodies used in analysis

As a service to our authors and readers, this journal provides supporting information supplied by the authors. Such materials are peer-reviewed and may be re-organized for online delivery, but are not copy-edited or typeset. Technical support issues arising from supporting information (other than missing files) should be addressed to the authors.
