# Supplementary material for: Glucocerebrosidase Mutations alter the endoplasmic reticulum and lysosomes in Lewy body disease
Source: J Neurochem. 2012 Oct;123(2):298–309. doi: 10.1111/j.1471-4159.2012.07879.x (PMC3494984; doi:10.1111/j.1471-4159.2012.07879.x)
Supplement: Supplementary file 1 [file jnc0123-0298-SD1.pdf]

Supplementary Information

Table ST1: Patient demographics of post mortem cohort

	**AAO-PD (years)**	**AAO-Dem (years)**	**Age-Death (years)**	**PM Delay (hours)**
PD wt (N=8)	72 ± 5	74 ± 6	77 ± 6	40 ± 24
PD mt (N=3)	70, 66,[a]	73, [c], 76	74 ± 4	30 ± 7
DLB wt (N=7)	70 ± 8	70 ± 5	76 ± 9	29 ± 25
DLB mt (N=4)	[b],52, 79, 75	66 ± 11	72 ± 11	43 ± 27
Con wt (N=11)	NA	NA	74 ± 15	18 ± 5
Con mt (N=5)	NA	NA	73 ± 18	36 ± 32

AAO-PD, age at onset of parkinsonism; AAO-Dem, age at onset of dementia; PM delay, post-mortem delay; wt, wild type (no mutation identified); mt, mutant. a, for case PD_3 age at onset of parkinsonism not known. b, for case DLB_1 no parkinsonian features reported. c, for case PD_2 no dementia features reported.

Table ST2: Primary antibodies used in analysis

**Primary antibodies**	**Source**	**Dilution**	**Catalogue Number**
Glucocerebrosidase	Sigma-Aldrich	1:1000	G4046
LAMP1	Developmental Studies Hybridoma Bank	1:1000	H4A3
LAMP2	Developmental Studies Hybridoma Bank	1:50,000	H4B4
Cathepsin D	Sigma-Aldrich	1:500,000	C0715
ATP13A2	Sigma-Aldrich	1:1000	A9607
BiP (HSPA5, GRP78)	Abnova	1:4000	PAB2462
GRP94	Cell Signaling Technology	1:1000	2104
HERP	Santa Cruz Biotechnology	1:500	19-Y: sc-100721
SCARB2/LIMP2	Sigma-Aldrich	1:500	HPA018014
α-synuclein	BD Transduction Laboratories	1:20,000	610786
GAPDH-HRP	Santa Cruz Biotechnology	1:4000	FL-335: sc-25778HRP

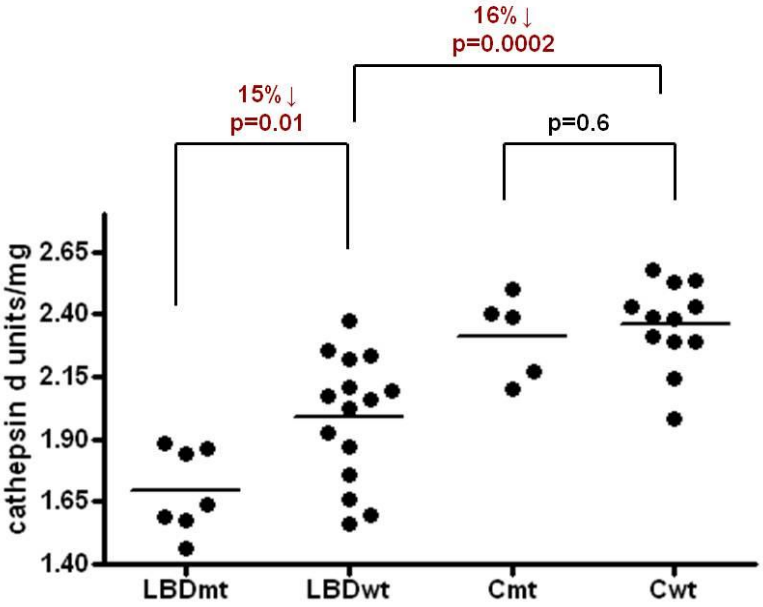

Supplementary Fig 1: Residual Cathepsin D activities in *GBA* mutation carriers: Samples of frontal cortex were assayed for Cathepsin D activity (LBDmt n=7, LBDwt n=15, Cmt n=5, Cwt n=12). The enzyme activities were calculated as values of nmoles of MCA released per minute per mg of enzyme sample. The protease activities were significantly reduced in LBD cases compared to control individuals (LBDwt vs. Cwt), and the depletion was aggravated by the presence of *GBA* mutation in LBD (LBDmt vs. LBDwt).

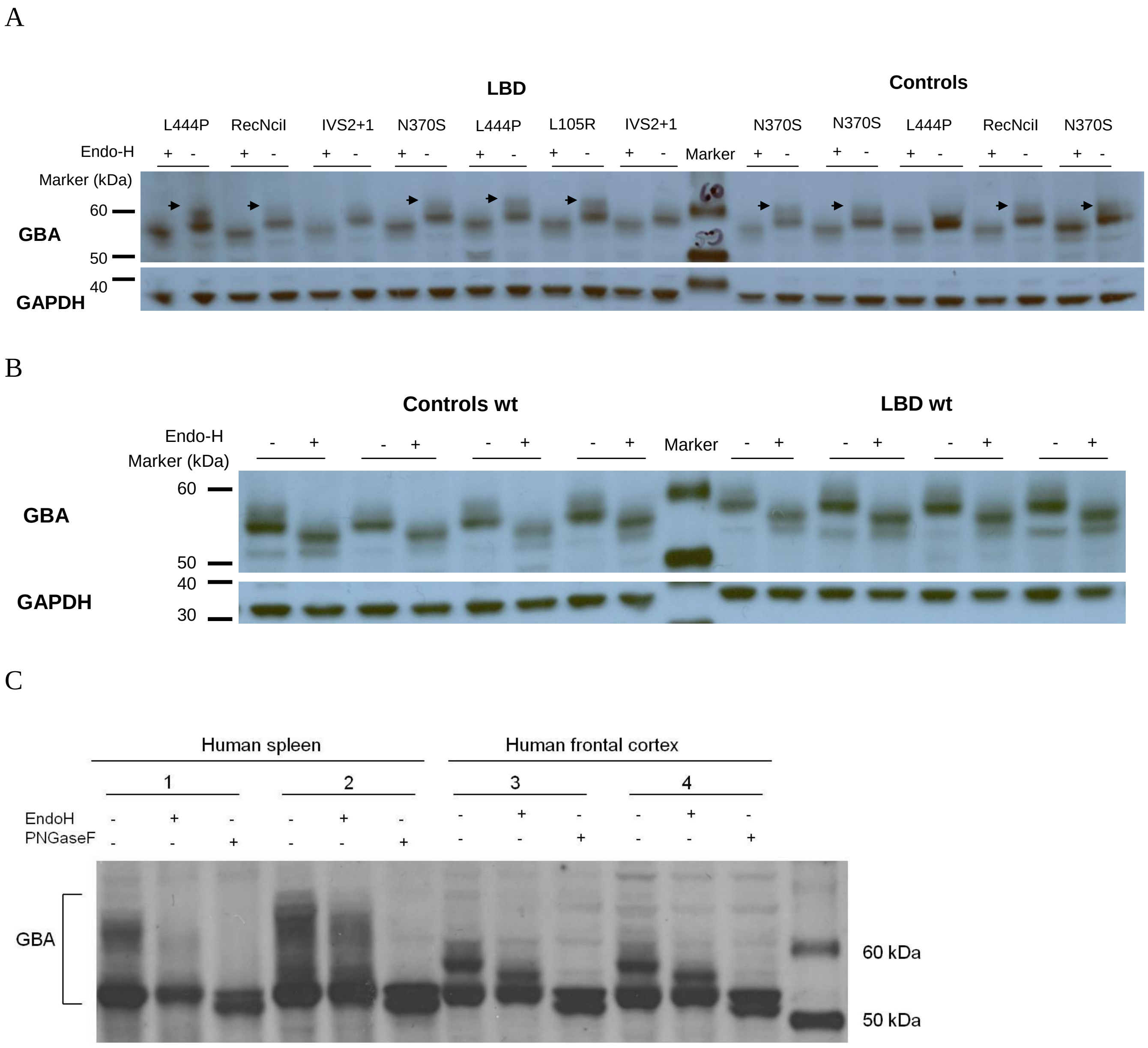

Supplementary Fig 2: Endoglycosidase-H sensitivity of glucocerebrosidase species in *GBA* mutation Carriers: Brain homogenates from individuals with *GBA* mutations were treated with (+) or without (-) Endoglycosidase-H to determine the presence of complex oligosaccharides and if there is abnormal folding and endoplasmic reticulum retention of glucocerebrosidase (GCase) protein. Endo-H treatment of brain GCase caused a large molecular weight shift following treatment suggesting that high order complex carbohydrates are not present to any great extent (A, B). Comparison of GCase from human brain and spleen Triton X-100 soluble fractions treated with Endo-H and PNGaseF reveals less extensive

glycosylation pattern (relative absence of the higher molecular weight over 60 kDa) and little resistance to Endo-H treatment in brain GCase (C).
